# Next-generation sequencing identified that RET variation associates with lymph node metastasis and the immune microenvironment in thyroid papillary carcinoma

**DOI:** 10.1186/s12902-024-01586-5

**Published:** 2024-05-11

**Authors:** Yongsheng Huang, Peiliang Lin, Jianwei Liao, Faya Liang, Ping Han, Sha Fu, Yuanling Jiang, Zhifan Yang, Ni Tan, Jinghua Huang, Renhui Chen, Nengtai Ouyang, Xiaoming Huang

**Affiliations:** 1 Cellular & Molecular Diagnostics Center, Sun Yat-sen Memorial Hospital, Sun Yat-sen University, Guangzhou, 510120 China; 2 Department of Otolaryngology-Head and Neck Surgery, Sun Yat-sen Memorial Hospital, Sun Yat-sen University, Guangzhou, 510120 China

**Keywords:** Next-generation sequencing, Thyroid carcinoma, Nodal metastasis, RET, Tumor microenvironment

## Abstract

**Background:**

To date, although most thyroid carcinoma (THCA) achieves an excellent prognosis, some patients experience a rapid progression episode, even with differentiated THCA. Nodal metastasis is an unfavorable predictor. Exploring the underlying mechanism may bring a deep insight into THCA.

**Methods:**

A total of 108 THCA from Chinese patients with next-generation sequencing (NGS) were recruited. It was used to explore the gene alteration spectrum of THCA and identify gene alterations related to nodal metastasis in papillary thyroid carcinoma (PTC). The Cancer Genome Atlas THCA cohort was further studied to elucidate the relationship between specific gene alterations and tumor microenvironment. A pathway enrichment analysis was used to explore the underlying mechanism.

**Results:**

Gene alteration was frequent in THCA. BRAF, RET, POLE, ATM, and BRCA1 were the five most common altered genes. RET variation was positively related to nodal metastasis in PTC. RET variation is associated with immune cell infiltration levels, including CD8 naïve, CD4 T and CD8 T cells, etc. Moreover, Step 3 and Step 4 of the cancer immunity cycle (CIC) were activated, whereas Step 6 was suppressed in PTC with RET variation. A pathway enrichment analysis showed that RET variation was associated with several immune-related pathways.

**Conclusion:**

RET variation is positively related to nodal metastasis in Chinese PTC, and anti-tumor immune response may play a role in nodal metastasis triggered by RET variation.

**Supplementary Information:**

The online version contains supplementary material available at 10.1186/s12902-024-01586-5.

## Introduction

As one of the most common malignant diseases, thyroid carcinoma (THCA) is the seventh malignancy diagnosed in women now [1]. In 2022, approximately 43,800 new cases were diagnosed in the United States [2]. Papillary thyroid carcinoma (PTC) is the main histologic type of THCA, which accounts for about 90% of THCA [3, 4]. Nodal metastasis could be up to 36% in PTC as reported in some studies, which is a predictor in estimating the survival of patients with THCA [5]. Although the prognosis is excellent for most THCA, which a 10-year survival rate of exceeds 90%, some patients experience a rapidly progressing episode and have an unfavorable prognosis, even with differentiated THCA [6]. For now, the underlying mechanism in the initiation and nodal metastasis of thyroid carcinoma is not clear.

At present, the mechanism of lymph node metastasis in PTC mainly focuses on basic research [7, 8]. A simple and convenient indicator for lymph node metastasis is very important for clinical applications. With the rapid development of sequencing, next-generation sequencing (NGS) sequencing has been widely used in the diagnosis of THCA [9, 10]. Most of the previous studies distinguish between benign and malignant THCA using NGS, including ThyroSeq, ThyroSeq v2, AmpliSeq Cancer Hotspot Panel v2, etc [10–13]. However, there is no extraordinary indicator to indicate lymph node metastasis of PTC.

In this study, we performed NGS in 108 patients with thyroid disease by a novel gene panel and identified RET variation is positively related to nodal metastasis in PTC. Moreover, RET variation is associated with immune cell infiltration levels and the cancer immunity cycle (CIC) activities. The underlying mechanism that RET is involved in several immune-related pathways may bring a deeper insight into THCA.

## Materials and Methods

### Patients

In this study, a total of 108 patients of the Sun Yat-sen University (SYSU) cohort were enrolled with patient-informed consent. The inclusion criteria are as follows: (1) the patient has a malignant thyroid tumor; (2) the malignant thyroid tumor was surgically removed, which pathological diagnosis and evaluable lymph node metastasis were available (3) tumor tissue performed NGS by the THCA 90-genes panel. Thyroid tumor tissue samples were collected from 108 patients between February 2021 and March 2022 at the Sun Yat-sen Memorial Hospital. The comprehensive clinical information (including age, gender, primary tumor sites, etc.) was collected retrospectively. The pathological diagnosis and lymph node metastasis of patients was performed by at least two professional pathologists. The pathological types of the patients mainly include papillary thyroid carcinoma (PTC), follicular thyroid carcinoma (FTC), medullary thyroid carcinoma (MTC), and anaplastic thyroid carcinoma (ATC).

### Next-generation sequencing (NGS)

Sequencing libraries were prepared using the THCA 90-genes panel (RigenBio). The THCA 90-gene panel is a targeted capture-based NGS for point mutations, insertions, deletions, and gene fusions (Table S1). Briefly, the DNA was extracted from thyroid formalin-fixed paraffin-embedded (FFPE) tumor tissues after the artificial estimation of tumor purity. An aliquot of DNA was fragmented, which was followed by end-repairing, a-tailing, ligation with a sequencing adapter, and adding unique dual indices. The purified libraries were subjected to targeted capture with the probes. Then the PCR amplification was performed to enrich captured libraries. The qualified enriched libraries were sequenced for 150 bp paired-end reads on NextSeq 550 sequencing system (Illumina, California, USA). As for bioinformatics analysis, the residual adapter sequences were removed by Trimmomatic (v0.38) [14]. Then the SNV/InDel was called by VarScan (v2.3.9) [15]. The variants were annotated by ANNOVAR [16, 17].

### Immune infiltration and CIC activity analysis

Validation data of THCA were downloaded from The Cancer Genome Atlas (TCGA) [18]. The somatic mutation data and gene expression profiles of THCA were downloaded by the Xena and cBioPortal [18, 19]. Patients with both mutation and gene expression data were further analyzed. The immune infiltration levels of THCA patients were acquired from TIMER2.0 [20, 21]. Various densities of immune cells of THCA patients were calculated by CIBERSORT [22]. The CIC is a series of stepwise events, including (Step 1) release of cancer cell antigens, (Step 2) cancer antigen presentation, (Step 3) priming and activation, (Step 4) trafficking of immune cells to tumors, (Step 5) infiltration of immune cells into tumors, (Step 6) recognition of cancer cells by T cells and (Step 7) killing of cancer cells [23]. We downloaded CIC activity scores of TCGA THCA samples from TIP [23]. In this study, a total of 487 PTC patients with genetic mutation data, clinical information, gene expression profiles, immune infiltration levels, various densities of immune cells, and CIC activity scores were obtained. Among these 487 PTC patients, 34 patients had RET variations: 33 with RET fusions, and one with RET mutation co-occurring with BRAF V600E mutation. According to RET variation, 487 PTC patients were divided into altered-RET (RET-MT, *n*=34) and RET wild-type (RET-WT, *n*=453) groups. Then, we divided the RET-WT samples into three groups: RAS mutated (RAS-MT, *n*=57), BRAF mutated (BRAF-MT, *n*=295), and other patients (*n*=101). We conduct subsequent immune-related analyses based on these groups.

### Protein-protein interaction analysis

The potential binding partners and co-expression molecules of RET were predicted by using the GeneMANIA database. In this way, a total of 20 potential binding partners and co-expression molecules were obtained for RET.

### Pathway enrichment analysis

For the underlying mechanisms of RET, a pathway enrichment analysis was performed. A total of 495 differentially expressed genes with |log (fold change) |>0.5 and *P*-value <0.05 were identified. Kyoto Encyclopedia of Genes and Genomes and Gene Ontology (KEGG) analysis was executed by DAVID [24, 25].

### Statistical analysis

We used R software and GraphPad Prism for statistical analyses. As for normally distributed continuous variables, the variables in binary groups were calculated by t-test. The non-normally distributed variables were calculated by the Mann-Whitney U test. The mutation frequency in different groups were analyzed by Chi-square or Fisher’s exact test. All statistical tests that *P* < 0.05 were considered statistical significance.

## Results

### Clinicopathologic characteristics of the included patients

Among 108 patients, most patients were young and female, which PTC was the main histologic type among. Seventy-eight patients confirmed lymph node metastasis in postoperative pathologic examination. Most of the patients were confirmed to be early-stage tumors finally (Table 1).

**Table 1:** The clinical factors of 108 thyroid cancer patients

Clinical characteristics	N
Total	108
Age	
<15	1
15-20	2
21-30	22
31-40	34
41-50	24
51-60	18
>60	7
Gender	
Female	65
Male	43
Histological type	
PTC	99
FTC	2
MTC	4
ATC	1
NA	2
Primary tumor site	
Left	32
Right	36
Isthmus	1
Bilateral	34
NA	5
Lymphatic metastasis	
Yes	78
No	30
Pathologic stage	
I	92
II-IV	12
X	2
NA	2
Pathologic T	
T1-T2	90
T3-T4	14
Tx	4
Pathologic N	
N0	30
N1	78
Pathologic M	
M0	108
M1	0

*PTC* Papillary thyroid carcinoma, *FTC* Follicular thyroid carcinoma, *MTC* Medullary thyroid carcinoma, *ATC* Anaplastic thyroid carcinoma, *NA* Not available

### Genetic variation landscape of thyroid disease

**Fig. 1:**
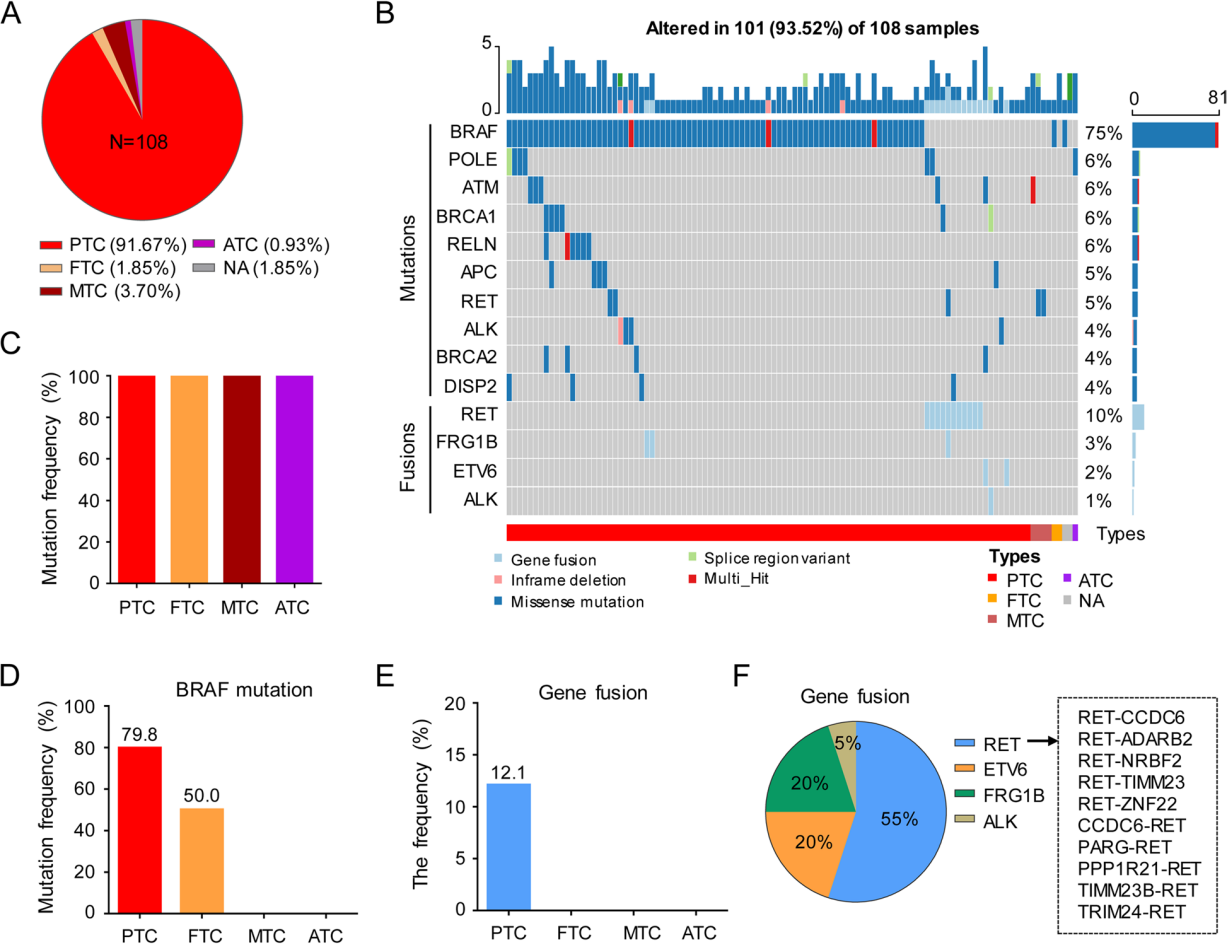
Genetic variation landscape of thyroid cancers. **A** The proportion of different tissue types in 108 patients. **B** The top 10 mutated genes and fusions in 108 patients. **C** The proportion of mutations in patients with different subtypes. **D** BRAF gene mutation frequency in patients with different subtypes. **E** Gene fusion frequency in patients with different subtypes. PTC, Papillary thyroid carcinoma; FTC, Follicular thyroid carcinoma; MTC, Medullary thyroid carcinoma; ATC, Anaplastic thyroid carcinoma; NA, Not available

Among the included patients, PTC made up 91.67% of the total 108 patients (Fig. 1A). The genetic mutation and gene fusion landscape of 108 patients was shown in Fig. 1B. All patients had gene alteration including mutation and gene fusion (Fig. 1C). The five most common mutated genes were BRAF, POLE, ATM, BRCA1, and RELN (Fig. 1B). Two MTC patients had NRAS mutations (p.Q61L and p.Q61K), while one FTC patient had NRAS mutations (p.Q61R) (Fig. S1A). Notably, the mutation frequency of RAS is low (2%) in PTC patients (Fig. S1B). TERT mutation in the cohort was not as frequent as reported in some other studies, which accounted for 3% of our included patients (data not shown). BRAF mutation was as high as 79.8% in PTC, whereas it was 50.0% in follicular thyroid carcinoma (Fig. 1D). Gene fusion was the most frequent in PTC, while no gene fusion was detected in other histologic types of THCA (Fig. 1E). Fusion of RET with other genes was the most frequent type of gene fusion in the included patients (Fig. 1F). Approximately 71% of PTC patients with gene fusion are younger than 30 years old (Fig. S2). Moreover, two PTC patients had RET point mutations, and the mutation frequency was approximately 50% (Fig. S3).

### RET variation is associated with lymph node metastasis

**Fig. 2:**
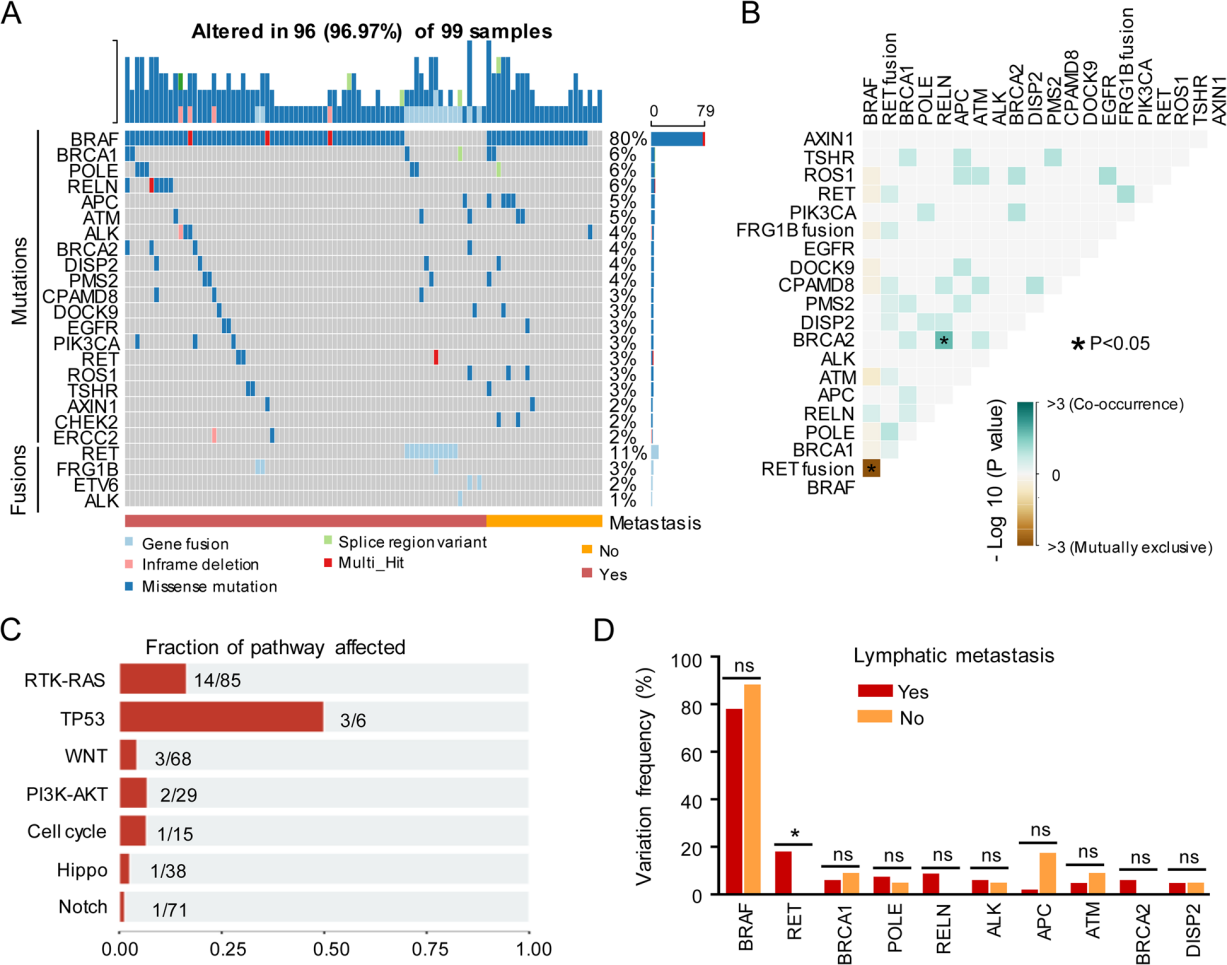
Genetic variation of patients with PTC. **A** The top 20 mutated genes and fusions in 99 PTC patients. **B** The cooccurrence of top 20 altered genes in PTC patients. **C** The fraction affected pathways of altered genes. **D** The variation frequency of top 20 altered genes in PTC patients with and without lymphatic metastasis. PTC, Papillary thyroid carcinoma; *, *P*<0.05; ns, no significance

As PTC was the dominant histologic type in the cohort, we made further analysis of the PTC subgroup. The top 10 frequent alterations of mutated genes and gene fusion are show in Fig. 2A. BRAF mutation occurred in about 80% of PTC. Co-occurrence analysis of the altered genes revealed that alteration of BRAF and RET fusion was mutually exclusive (Fig. 2B). However, BRCA2 mutation was positively associated with RELN mutation (Fig. 2B). Further analysis revealed that the altered genes affected seven different pathways (Fig. 2C). Mutation or gene fusion was detected in multiple genes on RTK-RAS, TP53, WNT, PI3K-AKT pathway in PTC, which RTK-RAS pathway was found to have the most molecular switches in PTC among (Fig. 2C).

As lymph node metastasis is common in PTC, which has a negative impact on survival, the molecular switches or alterations in samples from the patients with and without nodal metastasis were compared. The alteration frequency of RET was higher in the patients with lymph node metastasis than those without RET variation (Fig. 2D). However, there was no relationship between BRAF V600E mutation and lymph node metastasis in PTC (Fig. S4A). Although not statistically significant, an association between PTC with RET fusion and lymph node metastasis seems to exist (Fig. S4B). Next, we analyzed the relationship between the top 10 altered genes and lymph node metastasis and found that only RET variation was associated with lymph node metastasis (Fig. 2D). Moreover, APC variation was enriched in the patients without lymph node metastasis (Table S2).

### Pathologic features and tumor microenvironment (TME) in RET-MT and RET-WT PTC

**Fig. 3:**
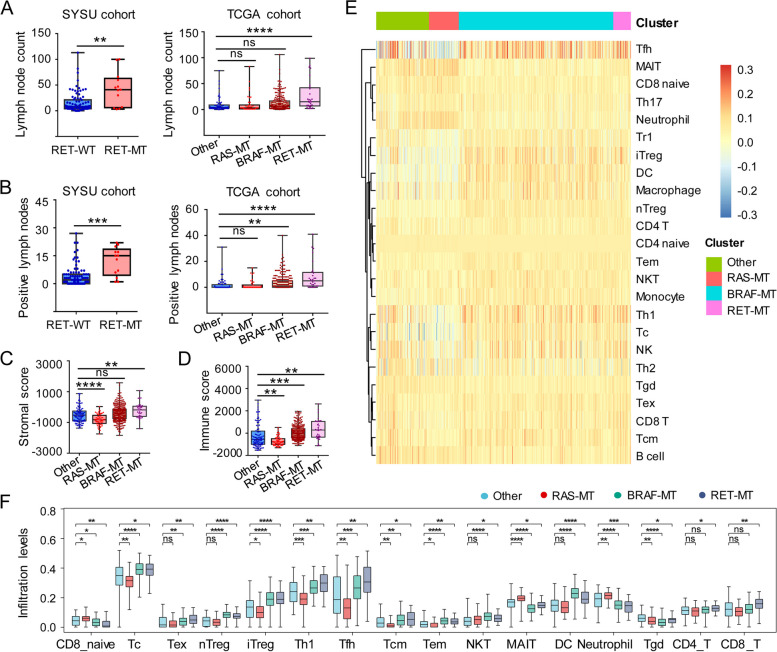
Pathologic features and TME in RET-MT and RET-WT PTC. **A** The lymph node examined counts in RET-MT and RET-WT PTC. **B** The positive lymph nodes in RET-MT and RET-WT PTC. **C** The stromal score in PTC patients among different groups in the TCGA cohort. D The immune score in PTC patients among different groups in the TCGA cohort. **E**, **F** The infiltration level of immune cells in PTC patients among different groups in the TCGA cohort. RET-MT, patients with RET variation; RET-WT, patients without RET variation; RAS-MT, patients with NRAS, KRAS, or HRAS mutation; BRAF-MT, patients with BRAF mutation; PTC, Papillary thyroid carcinoma; SYSU, Sun Yat-sen University; TCGA, The Cancer Genome Atlas. *, *P*<0.05; **, *P*<0.01; ***, *P*<0.001; ****, *P*<0.0001; ns, no significance

Next, the pathologic features and TME were compared between the patients with and without RET alteration in our cohort and the TCGA-THCA cohort. The clinicopathologic characteristics of the PTC patients with and without RET alteration was shown in Table S3. Notably, RET alteration is associated with age, pathologic T stage and N stage (Table S3). Moreover, both lymph node examined counts and positive lymph nodes were higher in the RET-MT group than the RET-WT group in our cohort (Fig. 3A, B). In the TCGA-THCA cohort, the RET-MT group also had more lymph node examined counts and positive lymph nodes than the other mutation group (Fig. 3A, B). However, there was no difference in the lymph node examined counts and positive lymph nodes between RAS-MT and the other mutation group (Fig. 3A, B). Compared with the other mutation group, BRAF-MT group only had a higher number of positive lymph nodes (Fig. 3A, B). This suggests that lymph node metastasis may be related to RET and BRAF mutation, but not RAS mutation. Indeed, the immune infiltration analysis revealed that both stromal and immune scores were higher in the RET-MT group than in the other mutation group (Fig. 3C, D). BRAF-MT group had higher immune scores (but not stromal scores) than the other mutation group (Fig. 3C, D). However, RAS-MT group had lower stromal and immune scores than the other mutation group (Fig. 3C, D). Further analysis showed that Tc, Tex, nTreg, iTreg, Th1, Tfh, Tcm, Tem, NKT, DC, NK, CD4 T, and CD8 T cells were recruited in PTC with RET alteration, while CD8 naïve, MAIT, Neutrophil, and Tgd were infertile (Fig. 3E, F).

**Fig. 4:**
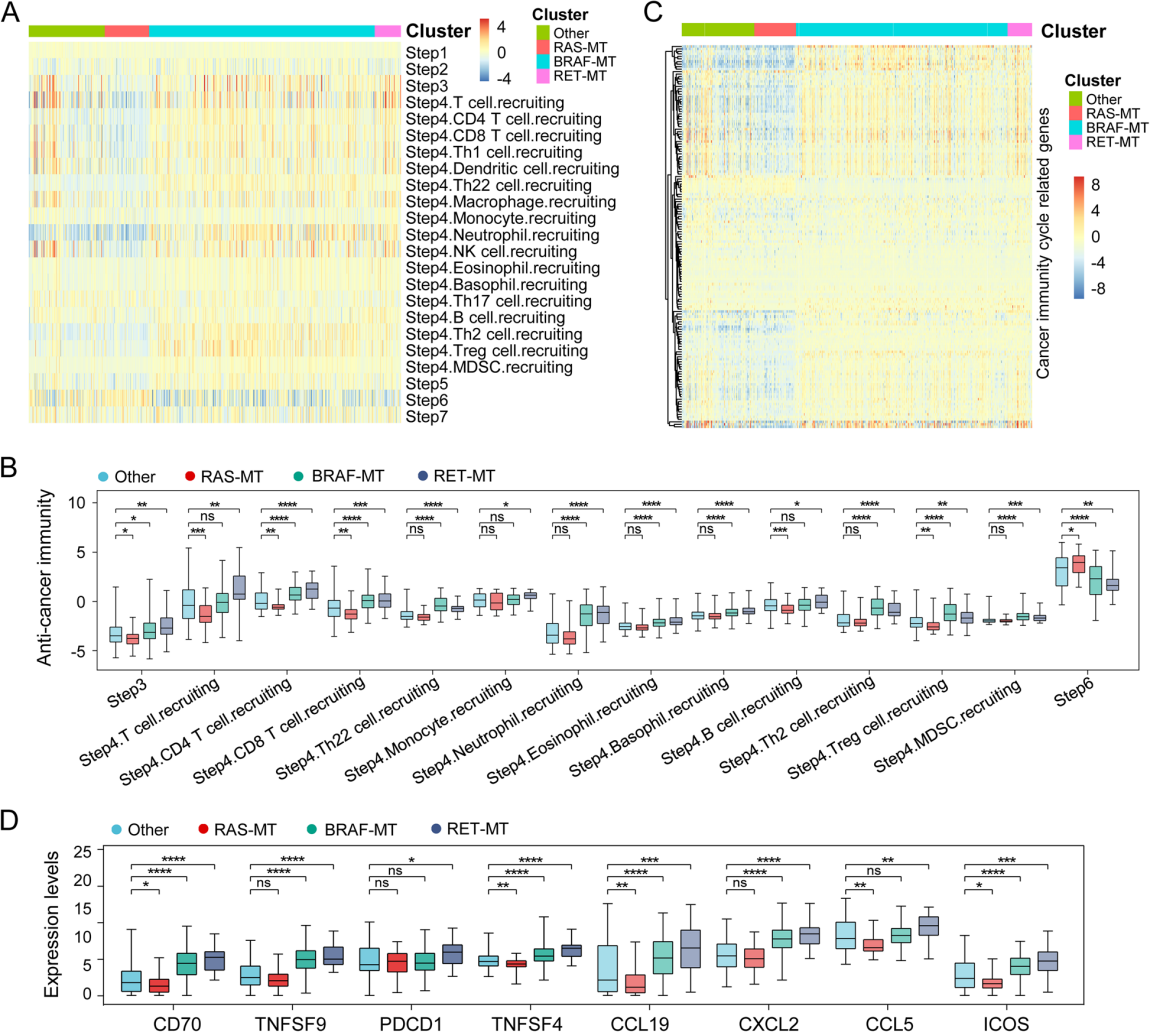
RET variation associated with the cancer immunity cycle activities. **A**, **B** The cancer immunity cycle activities among different groups in the TCGA cohort. **C** Heatmap of immune-related gene expression in PTC. **D** The expression of immune-related gene expression among different groups in the TCGA cohort. RET-MT, patients with RET variation; RAS-MT, patients with NRAS, KRAS, or HRAS mutation; BRAF-MT, patients with BRAF mutation; PTC, Papillary thyroid carcinoma; TCGA, The Cancer Genome Atlas. *, *P*<0.05; **, *P*<0.01; ***, *P*<0.001; ****, *P*<0.0001; ns, no significance

Furthermore, we performed CIC activities analysis and found that most of the steps, including priming and activation (Step 3) as well as trafficking of immune cells to tumors (Step 4) were activated in PTC with RET-MT or BRAF-MT, whereas recognition of cancer cells (Step 6) was suppressed (Fig. 4A, B). However, RAS-MT patients have the opposite situation, in which Step 3 and Step 4 were suppressed, whereas Step 6 was activated (Fig. 4A, B). The expression of several immune-related genes (including CD70, TNFSF4, CCL19, ICOS, etc.) in the RET-MT and BRAF-MT groups was up-regulated, but down-regulated in the RAS-MT group (Fig. 4C, D). These results suggest that RET alteration is associated with TME in PTC.

### RET alteration associated with cancer-related pathways

**Fig. 5:**
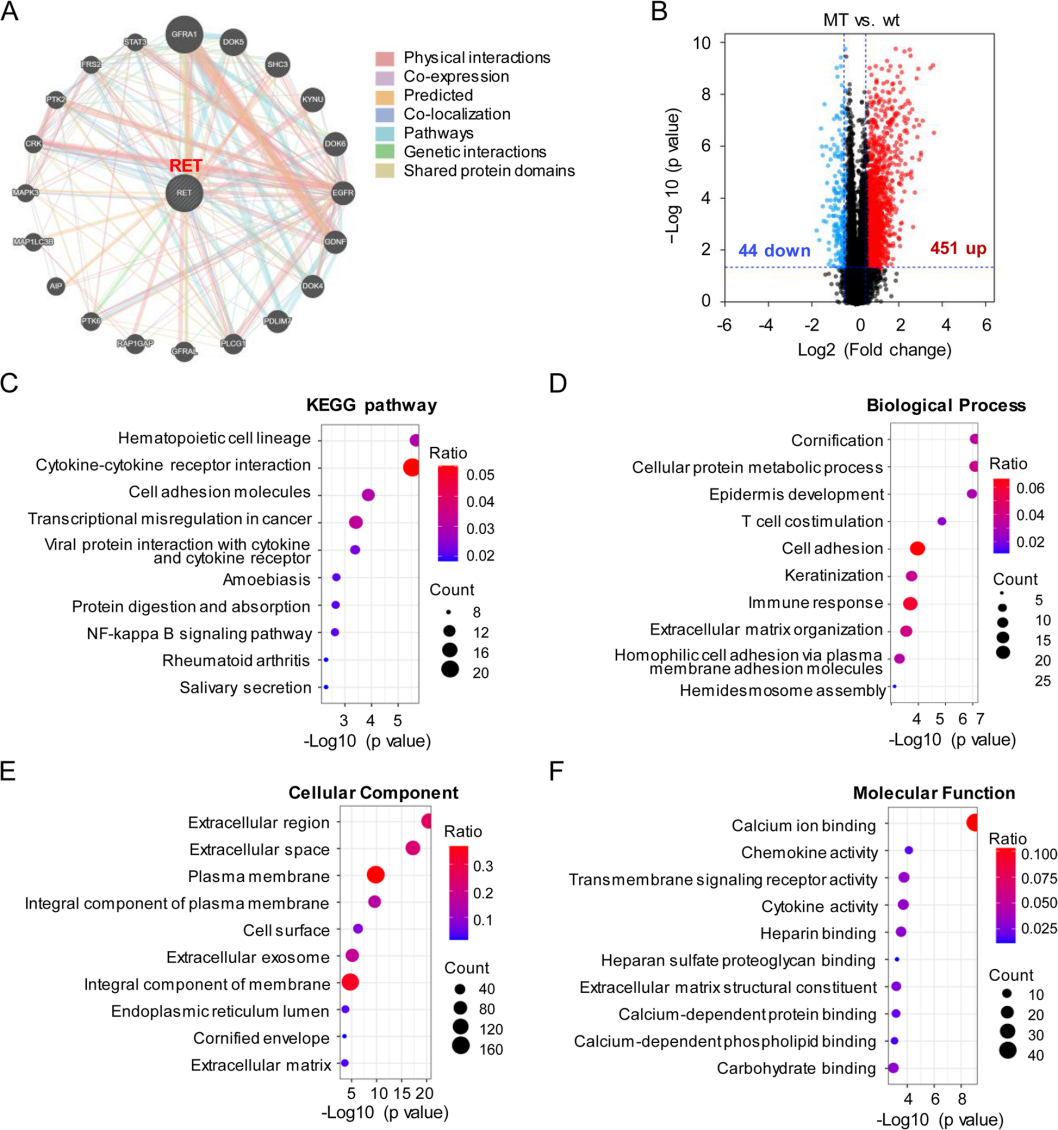
RET variation associated with cancer-related pathways. **A** Gene co-expression and interaction network of RET. **B** The volcano plot of DEGs (|Log2 fold change|>0.5, *P*<0.05) in altered-RET and RET wild-type PTC. **C** The DEGs enriched pathways. **D-F** GO analysis of the DEGs. DEGs, differentially expressed genes

To explore the potential mechanism between RET alteration and lymph node metastasis, we used the GeneMANIA database to explore the potential binding partners and co-expression molecules of RET. A total of 20 potential binding partners and co-expression molecules were obtained, including GFRA1, EGFR, STAT3, MAPK3, etc (Fig. 5A). Moreover, a pathway enrichment analysis revealed that the expression of 451genes was upregulated and that of 44 genes were downregulated in RET alteration groups (Fig. 5B). KEGG analysis showed that cytokine-cytokine receptor interaction, cell adhesion molecules, and transcriptional misregulation in cancer may play a role in nodal metastasis triggered by RET alteration (Fig. 5C). Abnormity of cellular protein metabolic process, T cell co-stimulation, cell adhesion, and immune response, may accelerate tumor progression in PTC (Fig. 5D-F).

## Discussion

Recently, genetic testing plays a vital role in the distinction between benign and malignant of thyroid disease. Numerous gene panel has been widely used in the diagnosis of THCA, including ThyroSeq, ThyroSeq v2, AmpliSeq Cancer Hotspot Panel v2, etc [10–13]. Nevertheless, the genetic profile and immune microenvironment of PTC with lymph nodal metastasis are not clear. In the context of clinical application, we have revealed the gene mutation characteristics of PTC by a novel gene panel and found that RET alteration is associated with lymph nodal metastasis in PTC. The TME and pathway enrichment analysis of RET-mutated tumors provides further insight into the molecular characteristics of PTC metastasis.

In most studies, RET rearrangements have been detected in human tumors, which are approximately 2.5-73% in PTC patients [26–28]. In this study, the frequency of RET rearrangements was 11% in PTC patients. Notably, RET rearrangement is mutually exclusive with BRAF mutation. This suggests that the role of RET in lymph node metastasis of PTC may be independent of BRAF mutation. To PTC, BRAF was the most frequent mutated gene [29, 30]. BRAF V600E has attracted much attention as one of the most common mutated genes in PTC. Indeed, many studies have found that PTC with BRAF V600E mutation is associated with lymph node metastasis [31, 32]. However, there are some inconsistent studies [33, 34]. For example, no association was established between BRAF V600E mutation and regional lymph node metastasis in PTC in Chinese patients [33]. In another Chinese cohort, there was no relationship between BRAF V600E mutation in lymph node metastasis and the number, extranidal extension, or stage of lymph node metastasis in PTC [34]. Here, we found that the BRAF V600E mutation was not associated with lymph node metastasis. Therefore, the relationship between BRAF V600E mutation and lymph node metastasis was inconsistent in different cohorts, which may be due to the different genetic backgrounds of different races.

In our study, RET alteration was associated with nodal metastasis and TME in PTC. The patients, who obtain RET alteration, have different tumor-infiltrating lymphocyte subpopulations. Specifically, several immune cells (including Tc, Tex, nTreg, iTreg, etc.) were recruited in PTC with RET alteration, while CD8 naïve, MAIT, Neutrophil, and Tgd were infertile (Fig. 3E, F). Furthermore, most of the CIC steps, including priming and activation (Step 3) as well as trafficking of immune cells to tumors (Step 4) were activated in PTC with RET alteration, whereas recognition of cancer cells (Step 6) was suppressed (Fig. 4A, B). However, RAS-MT patients have the opposite situation in TME, including stromal and immune scores, immune cell infiltration levels, CIC activities, and the expression of immune-related genes. These suggest that lymph node metastasis may be related to RET and BRAF mutation, but not RAS mutation. Indeed, there was no difference in the lymph node examined counts and positive lymph nodes between RAS-MT and the other mutation group (Fig. 3A, B). The tumor immune disorder, and excessive recruitment of lymphocytes but depressive cancer cell recognition, led to immunodepleting and facilitated tumor immune escape, which may make contributions to nodal metastasis.

THCA is one of the most prone tumors to gene fusion. Therefore, gene fusion has become a tumor diagnostic tool for THCA. In our study, gene fusion only be detected in PTC (Fig. 1E). This suggests that in thyroid benign and malignant detection, gene fusion may not be suitable for FTC, MTC or ATC. In this case, the detection of gene fusion based on cell morphology may improve the diagnostic efficiency of THCA. Fortunately, our gene panel included several common fusion genes for THCA (such as RET, ALK, ROS1, IGF2BP3, NTRK1/2/3, PPARG, etc.) as well as 89 THCA-associated gene variants. Therefore, compared to single gene mutation and gene fusion detection, our panel is suitable for all types of THCA. Gene fusion can occur either in upstream or downstream of the gene. As previously reported, the mainly RET fusions are CCDC6-RET and NCOA4-RET in PTC [26, 35]. Here, in addition to CCDC6-RET, we also detected other forms of RET fusions, including PARG-RET, PPP1R21-RET, TIMM23B-RET, etc (Fig. 1F). Unfortunately, in our internal cohort of 108 patients, we did not detect the NCOA4-RET fusion, which may be due to the small sample size. Except for the fusion that occurred in upstream of RET, several other forms of gene fusion in RET downstream were also detected, such as RET-CCDC6, RET-NRBF2, RET-ZNF22, etc (Fig. 1F). Generally, the fusion that occurred in upstream of RET resulted in extremely activation of RET and further promote the progression of PTC [36]. However, the function of gene fusion occurring in downstream of RET in PTC needs further study.

In the germline, point mutations of RET are responsible for multiple endocrine neoplasia type 2 and familial medullary thyroid carcinoma [37]. RET somatic point mutations are rare in PTC patients. In this study, two PTC patients had RET point mutations, and the mutation frequency was approximately 50% (Fig. S3). This suggests that these RET point mutations are most likely a germline variation. Furthermore, although RET point mutation co-occurs with BRAF V600E mutation, the mutation abundance of BRAF V600E was significantly lower than that of RET point mutation, suggesting that a large proportion of tumor cells have only RET point mutation but no BRAF V600E mutation. Indeed, other researchers found that there was no relationship between BRAF V600E mutation and lymph node metastasis in PTC [33, 34]. These suggest that lymph node metastasis may not be caused by the group of PTC cells with BRAF V600E, but is more likely to be caused by the group of PTC cells with only RET point mutations. Of course, further research is needed to confirm this.

Currently, more and more inhibitors targeting RET variation are used in the treatment of human cancers [38–44]. For example, selpercatinib, a selective RET inhibitor designed to inhibit diverse RET fusions, was used in the thyroid, lung, and other cancers [43, 44]. We and others found that the variation form of the RET gene in the thyroid is mainly gene fusion. This suggests that selpercatinib is an ideal choice for THCA with RET fusion. Indeed, a phase 1-2 clinical trial showed that selpercatinib presents durable efficacy with low-grade toxic effects in patients with medullary thyroid carcinoma [45]. However, in addition to gene fusion, missense mutations of RET also occur in THCA. Fortunately, ponatinib (AP24534) is a novel potent inhibitor of oncogenic RET mutants associated with THCA [46]. Therefore, we can choose different RET inhibitors based on the mutation types. In clinical practice, a single RET inhibitor may not be effective for patients. Therefore, patients are usually given a combination of drugs. Unfortunately, the combination of RET inhibitors and other targeted drugs is easy to produce resistance. Here, we found that RET variation is associated with TME and CIC activities. Moreover, several immune checkpoints associated genes were upregulated in PTC with RET alteration. This suggests that RET inhibitors plus immunotherapy may be the ideal treatment for malignant tumors, which certainly need further clinical studies.

Lymph node metastasis is a crucial factor in the PTC progression. The distribution of immune subsets in tumor-infiltrating lymphocytes differed in with and without lymph node metastasis of PTC. The profiles of PTC without nodal metastasis correlate with a more favorable biology for the host. Indeed, KEGG analysis showed that cytokine-cytokine receptor interaction and cell adhesion molecules were enriched in PTC patients with RET alteration. RET alteration results in the interaction changes between tumor cells and the host, which trigger the nodal metastasis of PTC. Furthermore, abnormity of cellular protein metabolic process, T cell co-stimulation, cell adhesion, and immune response, may accelerate the metastasis progression in PTC. PTC has many morphologic subtypes, exhibiting different mutational, clinicopathological, and prognostic characteristics [3], which need to be considered in PTC research. Although we provided the genetic mutation profiles of PTC, more detailed subtype information is not available in our study. Therefore, the present findings may be totally or in part driven by specific PTC subtypes that are not assessable in the present cohort. Through comprehensive analysis, the molecular characteristics of PTC with and without node metastasis were characterized by a gene panel, which may guide significance for clinical application.

## Conclusions

In summary, NGS identified RET variation is positively related to nodal metastasis in Chinese PTC. RET variation is associated with pathologic features (pathologic T stage and N stage), immune cell infiltration levels (such as NK, CD4 T, and CD8 T cells, etc.), CIC activities (Step3, Step4, Step6), and the expression of immune checkpoints associated genes (such as CD70, TNFSF4, CCL19, ICOS, etc.). Moreover, RET is involved in several immune-related pathways. The understanding of the molecular characteristics and microenvironment of RET-induced metastasis further promotes the clinical application of NGS in PTC.

### Supplementary Information

**Supplementary Material 1.**

**Supplementary Material 2.**

## Data Availability

The datasets generated and/or analyzed during the current study are available in the Supplementary Material.
