# Supplementary material for: Next-generation sequencing identified that RET variation associates with lymph node metastasis and the immune microenvironment in thyroid papillary carcinoma
Source: BMC Endocr Disord. 2024 May 11;24:68. doi: 10.1186/s12902-024-01586-5 (PMC11088169; doi:10.1186/s12902-024-01586-5)
Supplement: Supplementary file 1 — Supplementary Material 1. [file 12902_2024_1586_MOESM1_ESM.pdf]

Supplementary data

Supplementary Tables

Table S1. Gene list of the thyroid cancer 90-genes panel

SNV/InDel (89)				Fusion Kinase Domain (9)
AKT1	DOCK9	MEN1	ROS1	ALK
ALK	DPYD	MET	SF3B1	BRAF
APC	EGFR	MLH1	SMAD4	IGF2BP3
ATM	EIF1AX	MSH2	SMARCA4	NTRK1
AXIN1	EPCAM	MSH6	SPOP	NTRK2
BMPR1A	ERBB2	MUTYH	STK11	NTRK3
BRAF	ERCC1	NF1	TERT	PPARG
BRCA1	ERCC2	NF2	TIRAP	RET
BRCA2	ETV6	NRAS	TP53	ROS1
CCNE1	EZH1	NRG3	TSHR	
CDH1	FBXW7	NTRK1	UGT1A1	
CDK4	FGFR1	NTRK2	VHL	
CDK6	FRG1BP	PBRM1	XRCC1	
CDKN2A	GNAS	PDGFRA	ZNF148	
CDKN2B	GREM1	PIK3CA		
CDKN2C	GSTP1	PMS2		
CHEK1	HDAC2	POLE		
CHEK2	HRAS	PPARG		
CPAMD8	IDH1	PPM1D		
CTNNB1	JAK2	PTEN		
CYP1B1	KDM6B	PTPN11		
CYP2D6	KIT	RB1		
DDX3X	KRAS	RBM10		
DICER1	MAP2K1	RELN		
DISP2	MDC1	RET		

Table S2. Top 10 gene variations of 99 PTC patients with and without lymph node metastasis

Gene variation	N	Metastasis	Non- metastasis	P
Total	99	75	24	
BRAF				0.3861
WT	20	17	3	
MT	79	58	21	
RET				0.0341
WT	86	62	24	
MT	13	13	0	
BRCA1				0.6303
WT	93	71	22	
MT	6	4	2	
POLE				1
WT	93	70	23	
MT	6	5	1	
RELN				0.331
WT	93	69	24	
MT	6	6	0	
ALK				1
WT		71	23	
MT		4	1	
APC				0.0117
WT	94	74	20	
MT	5	1	4	
ATM				0.5921
WT	94	72	22	
MT	5	3	2	
BRCA2				0.5695
WT	95	71	24	
MT	4	4	0	
DISP2				1
WT	95	72	23	
MT	4	3	1	

WT, Wild type; MT, mutation and gene fusion.

Table S3. The clinical factors of 99 PTC patients

		RET status		
Clinical characteristics	N	WT	Variation	P
Total	99	86	13	
Age				
<15	1	0	1	0.001
15-20	2	1	1	
21-30	20	13	7	
31-40	32	31	1	
41-50	22	21	1	
51-60	17	15	2	
>60	5	5	0	
Gender				0.7611
Female	61	52	9	
Male	38	34	4	
Primary tumor site				0.6335
Left	27	24	3	
Right	36	32	4	
Bilateral	32	26	6	
NA	4	4	0	
Pathologic stage				0.8229
I	87	75	12	
II-IV	9	8	1	
NA	3	3	0	
Pathologic T				0.0132
T1+T2	83	75	8	
T3+T4	13	8	5	
Tx	3	3	0	
Pathologic N				0.0286
N0	24	24	0	
N1	75	62	13	
Pathologic M				
M0	99	86	13	
M1	0	0	0	

Supplementary Figures

A

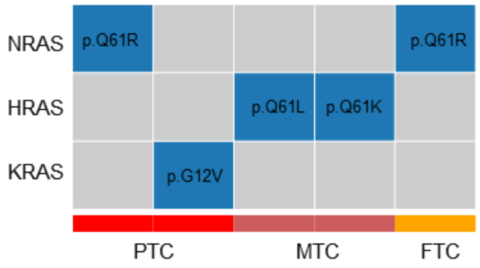

B

Mutation frequency

	NRAS	HRAS	KRAS
PTC (n=99)	1%	0	1%
MTC (n=4)	0	50%	0
FTC (n=2)	50%	0	0

Figure S1. RAS mutation in thyroid cancers

(A) Amino acid changes of RAS in patients. (B) The mutation frequency of RAS in patients with different subtypes. PTC, Papillary thyroid carcinoma; FTC, Follicular thyroid carcinoma; MTC, Medullary thyroid carcinoma.

A

Sample_ID	gene	Age	Age_range
SYSUTN00995	ALK	21	21-30
SYSUM0045363	ETV6	60	51-60
SYSUTN00784	ETV6	27	21-30
SYSUTN00097	FRG1B	25	21-30
SYSUTN00110	FRG1B	39	31-40
SYSUTN00139	FRG1B	44	41-50
SYSUM0030203	RET	28	21-30
SYSUM0030419	RET	23	21-30
SYSUM0031112	RET	19	15-20
SYSUM0039094	RET	36	31-40
SYSUM0047359	RET	26	21-30
SYSUM0052794	RET	27	21-30
SYSUM0056982	RET	29	21-30
SYSUTN00097	RET	25	21-30
SYSUTN00262	RET	23	21-30
SYSUTN01360	RET	53	51-60
SYSUY0521	RET	12	<15

B

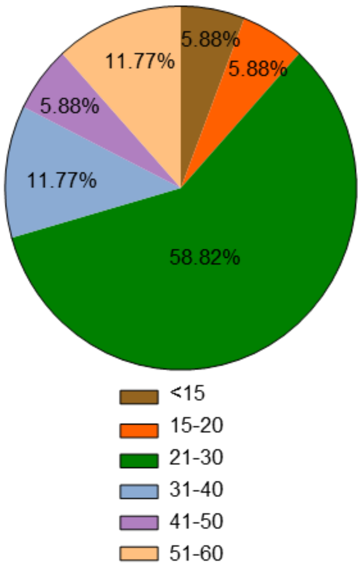

Figure S2. Distribution of gene fusion at different ages in PTC

(A) Gene fusion and age information in PTC patients. (B) The percentage of gene fusion among different age groups. PTC, Papillary thyroid carcinoma.

Sample_ID	Gene	Chr	HGVSp	Mutation Frequency
SYSUM0033266	BRAF	chr7	NP_004324.2:p.V600E	17.63%
SYSUM0033266	RET	chr10	NP_066124.1:p.V939I	48.97%
SYSUM0056983	BRAF	chr7	NP_004324.2:p.V600E	37.48%
SYSUM0056983	RET	chr10	NP_066124.1:p.S1021P	52.24%

Figure S3. RET point mutation and BRAF mutation information in two PTC patients.

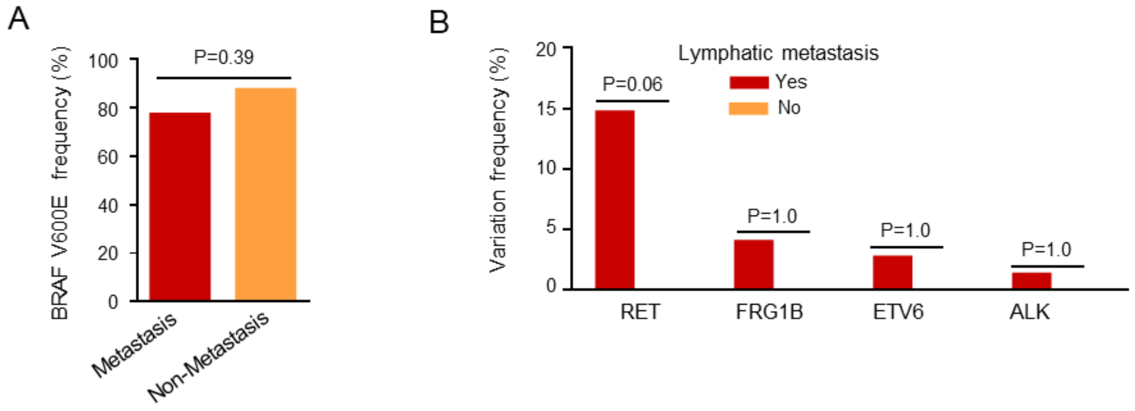

Figure S4. Gene variation frequency in PTC with lymphatic metastasis and lymphatic without metastasis

(A) The BRAF V600E mutation frequency in PTC with lymphatic metastasis and lymphatic without metastasis. (B) The gene fusion variation frequency in PTC with lymphatic metastasis and lymphatic without metastasis. PTC, Papillary thyroid carcinoma; ns, no significance.
